# Hematological indicator-based machine learning models for preoperative prediction of lymph node metastasis in cervical cancer

**DOI:** 10.3389/fonc.2024.1400109

**Published:** 2024-08-13

**Authors:** Huan Zhao, Yuling Wang, Yilin Sun, Yongqiang Wang, Bo Shi, Jian Liu, Sai Zhang

**Affiliations:** ^1^ School of Medical Imaging, Bengbu Medical University, Bengbu, Anhui, China; ^2^ Department of Gynecology and Oncology, First Affiliated Hospital, Bengbu Medical University, Bengbu, Anhui, China

**Keywords:** cervical cancer, lymph node metastasis, machine learning, hematological indicators, preoperative prediction

## Abstract

**Background:**

Lymph node metastasis (LNM) is an important prognostic factor for cervical cancer (CC) and determines the treatment strategy. Hematological indicators have been reported as being useful biomarkers for the prognosis of a variety of cancers. This study aimed to evaluate the feasibility of machine learning models characterized by preoperative hematological indicators to predict the LNM status of CC patients before surgery.

**Methods:**

The clinical data of 236 patients with pathologically confirmed CC were retrospectively analyzed at the Gynecology Oncology Department of the First Affiliated Hospital of Bengbu Medical University from November 2020 to August 2022. The least absolute shrinkage and selection operator (LASSO) was used to select 21 features from 35 hematological indicators and for the construction of 6 machine learning predictive models, including Adaptive Boosting (AdaBoost), Gaussian Naive Bayes (GNB), and Logistic Regression (LR), as well as Random Forest (RF), Support Vector Machines (SVM), and Extreme Gradient Boosting (XGBoost). Evaluation metrics of predictive models included the area under the receiver operating characteristic curve (AUC), accuracy, specificity, sensitivity, and F1-score.

**Results:**

RF has the best overall predictive performance for ten-fold cross-validation in the training set. The specific performance indicators of RF were AUC (0.910, 95% confidence interval [CI]: 0.820–1.000), accuracy (0.831, 95% CI: 0.702–0.960), specificity (0.835, 95% CI: 0.708–0.962), sensitivity (0.831, 95% CI: 0.702–0.960), and F1-score (0.829, 95% CI: 0.696–0.962). RF had the highest AUC in the testing set (AUC = 0.854).

**Conclusion:**

RF based on preoperative hematological indicators that are easily available in clinical practice showed superior performance in the preoperative prediction of CC LNM. However, investigations on larger external cohorts of patients are required for further validation of our findings.

## Introduction

Cervical cancer (CC) is one of the most common gynecological malignancies, with 600,000 new cases and 340,000 deaths reported worldwide in 2020 (1). Multiple studies have demonstrated that lymph node metastasis is an important independent risk factor affecting the prognosis of patients with CC and remains the major cause of mortality in CC patients (2, 3). The 5-year overall survival rate of CC patients without LNM is 80–90%, whereas in those patients with LNM, it is reduced to 50–65% (4–6). Therefore, the 2018 International Federation of Gynecology and Obstetrics (FIGO) officially incorporated LNM into the CC staging system (7). The importance of LNM in the diagnosis, treatment decision and prognosis assessment of CC is increasing. For early-stage CC patients without LNM, radical hysterectomy is recommended (8); for CC patients with LNM, radiotherapy or chemotherapy is the recommended treatment (9). Therefore, the accurate preoperative evaluation of LNM status in CC patients is essential for treatment decisions and prognostic assessment.

Lymph nodes biopsy is the gold standard for diagnosing LNM status (10); however, it is invasive and can cause complications, such as pain and lymphedema (11). Currently, imaging examination is a conventional diagnostic method for the preoperative and noninvasive evaluation of LNM status. Common imaging examinations include computed tomography (CT), magnetic resonance imaging (MRI), and positron emission tomography-CT (PET-CT) (10, 12). However, the detection of metastatic lymph nodes via CT and MRI mainly relies on morphological criteria and has relatively low sensitivity (38–56%) (13). Although PET-CT is considered the most effective method for detecting CC LNM, it has a high false-positive rate (14–16). By challenging the limits of traditional imaging examinations, emerging radiomics can further improve the accuracy of preoperative prediction of CC LNM (17, 18). However, the current research on radiomics for the preoperative prediction of CC LNM is still in its initial stages, and there is still a gap in knowledge from a practical application standpoint.

In recent years, with the development of artificial intelligence technology, machine learning (ML) has been playing an increasingly important role in the identification of LNM status in a variety of cancers, including breast cancer, kidney cancer, colon cancer, lung cancer, and cervical cancer (19–23). For example, Arezzo et al. (23) developed an Extreme Gradient Boosting (XGBoost) model based on clinical features and pelvic MRI features for the prediction of LNM in patients with advanced CC. The results of the study showed that the XGBoost model exhibited good predictive performance (89% accuracy, 83% precision, 83% recall, 0.79 AUC). Yu et al. (19) used the Random Forest (RF) algorithm to select MRI radiomics features and establish a Support Vector Machines (SVM) model for predicting axillary lymph node status in breast cancer. The results showed that the AUC of SVM in the training cohort and the external validation cohort were 0.90 and 0.91, respectively. All of the above studies show that ML models have some potential in predicting cancer LNM status.

Hematological indicators are quantifiable indicators that are clinically accessible. Previous studies have suggested associations between some hematological indicators and CC LNM. For example, increased preoperative plasma squamous cell carcinoma antigen (SCC-Ag) levels may predict an increased incidence of CC LNM (24, 25). Moreover, Gavrilescu et al. (26) demonstrated that CC patients without LNM had a significantly higher neutrophil-lymphocyte ratio (NLR) than CC patients with LNM. To our knowledge, no studies have used pure hematological indicators to build machine learning models for the preoperative prediction of LNM status in CC patients. Therefore, this study aimed to evaluate the feasibility of machine learning models characterized by preoperative hematological indicators to predict the LNM status of CC patients before surgery.

## Methods

### Participant characteristics

The clinical data of CC patients who were admitted to the Department of Gynecology and Oncology of the First Affiliated Hospital of Bengbu Medical University (Anhui, China) from November 2020 to April 2021 were retrospectively analyzed. The inclusion criteria were as follows: (1) patients who were first diagnosed with CC; (2) in line with the indication of CC radical surgery, radical hysterectomy and pelvic lymph node dissection were performed; and (3) patients with CC that were confirmed via postoperative pathology. The exclusion criteria were as follows: (1) patients complicated with other malignancies; and (2) patients with missing clinical and pathological data.

This retrospective study was approved by the Clinical Medical Research Ethics Committee of The First Affiliated Hospital of Bengbu Medical University (Bengbu, Anhui, China) (registration number: 2021KY010). The experiments were performed in strict accordance with the ethical standards laid down in the 1964 Declaration of Helsinki and its later amendments. Written inform consent was waived by the Clinical Medical Research Ethics Committee of The First Affiliated Hospital of Bengbu Medical University.

### Data collection and feature selection

Clinical features and hematological indicators were collected from the clinical data for patients with CC. Hematological indicators included routine blood indicators, routine biochemical indicators, coagulation function indicators, and tumor markers. Routine blood indicators included white blood cell (WBC), percentage of neutrophil (NEUT %), percentage of lymphocyte (LYM %), percentage of monocytes (MON %), hemoglobin (HGB), platelet large cell ratio (PLCR), etc.; routine biochemical indicators included alanine aminotransferase (ALT), aspartate aminotransferase (AST), prealbumin (PAB), total protein (TP), albumin (ALB), globulin (GLB), total cholesterol (TCHO), low-density lipoprotein (LDL), Cystatin C (Cys C), c-reactive protein (CRP), superoxide dismutase (SOD), etc.; coagulation function indicators included prothrombin time (PT), fibrinogen (FIB), D-dimer (DD), thrombin time (TT), activated partial thromboplastin time (APTT), international normal ratio of prothrombin time (PT-INR), and prothrombin activity (PTA); tumor markers included squamous cell carcinoma antigen (SCC-Ag). Various hematological indicators were measured by using a BC-6000plus automated hematology analyzer (Mindray, Shenzhen, China), a Sysmex CS5100 automatic blood coagulation analyzer (Sysmex, Kobe, Honshu Island, Japan), and an automatic biochemical analyzer (MEDATC, Shanghai, China).

### Data set division and data class balance

The training set and the testing set were randomly generated in a ratio of 7:3. However, there is an imbalance of sample categories in the training dataset (21% of the samples with LNM and 79% of the samples without LNM), which can lead to a large bias in the classification results of the machine learning models (27). Currently common class balancing methods include random oversampling, random undersampling and synthetic sampling methods. Both random oversampling and random undersampling can balance the distribution of sample classes in the dataset, which is conducive to alleviating the data imbalance problem. However, random oversampling will repeat a few class samples in the dataset many times, which can easily lead to overfitting of the model; random undersampling will remove some samples in the dataset, which leads to the problem of information loss. Synthetic sampling methods are an improvement on random sampling methods, and the most classic and popular synthetic sampling method is the synthetic minority over-sampling technique (SMOTE) (28). This method can effectively reduce the overfitting of the model and enhance the generalization ability of the model by randomly constructing non-repeating samples on the connecting lines of the same few classes of samples. SMOTE can compensate for the shortcomings of random oversampling to some extent. Therefore, in this study, the SMOTE method was used to class balance the training set prior to feature selection.

### Feature selection

In biological data, the performance of various machine learning classifiers depends heavily on the selection of important features. The methods of feature selection are categorized into rank-based and subset methods (29). Ranking-based feature selection methods do not depend on the performance of the algorithm, are computationally fast and less prone to overfitting, and can rank the importance of all features. Popular ranking-based methods include information gain, Fisher score, chi-square and minimum redundancy maximum relevance (30). However, ranking-based methods do not consider the joint importance of features and lack a threshold to determine the optimal number of features. Therefore, the ranking-based feature selection method was not selected for this study. Subset methods are feature selection methods that determine thresholds based on certain criteria to select the optimal subset of features (31). Popular subset-based methods include the least absolute shrinkage and selection operator (LASSO) and Recursive Feature Elimination (RFE) (32). However, RFE is a feature selection method based on a particular machine learning model (such as XGBoost, RF, and SVM). In order to avoid the influence of the basic model used for RFE on the results of the study, only the LASSO method was used for feature selection in this study.

### Establishment and evaluation of machine learning models

Following the recommendations made by the Scikit-Learn developers, we used six supervised machine learning models to predict CC LNM. The six machine learning models were Adaptive Boosting (AdaBoost), Gaussian Naive Bayes (GNB), Logistic Regression (LR), RF, SVM and XGBoost.

In this study, accuracy, specificity, sensitivity, F1-score and the areas under the receiver operator characteristic curves (AUC) were used as assessment metrics to compare the performance of the models. The ten-fold cross-validation was performed in the training set, and the AUC of the ten-fold cross-validation was used as the main evaluation metric to identify the machine learning model with the best prediction performance. This study evaluates the prediction performance of six machine learning models in the testing set using the receiver operating characteristic (ROC) curves.

**Figure 1:**
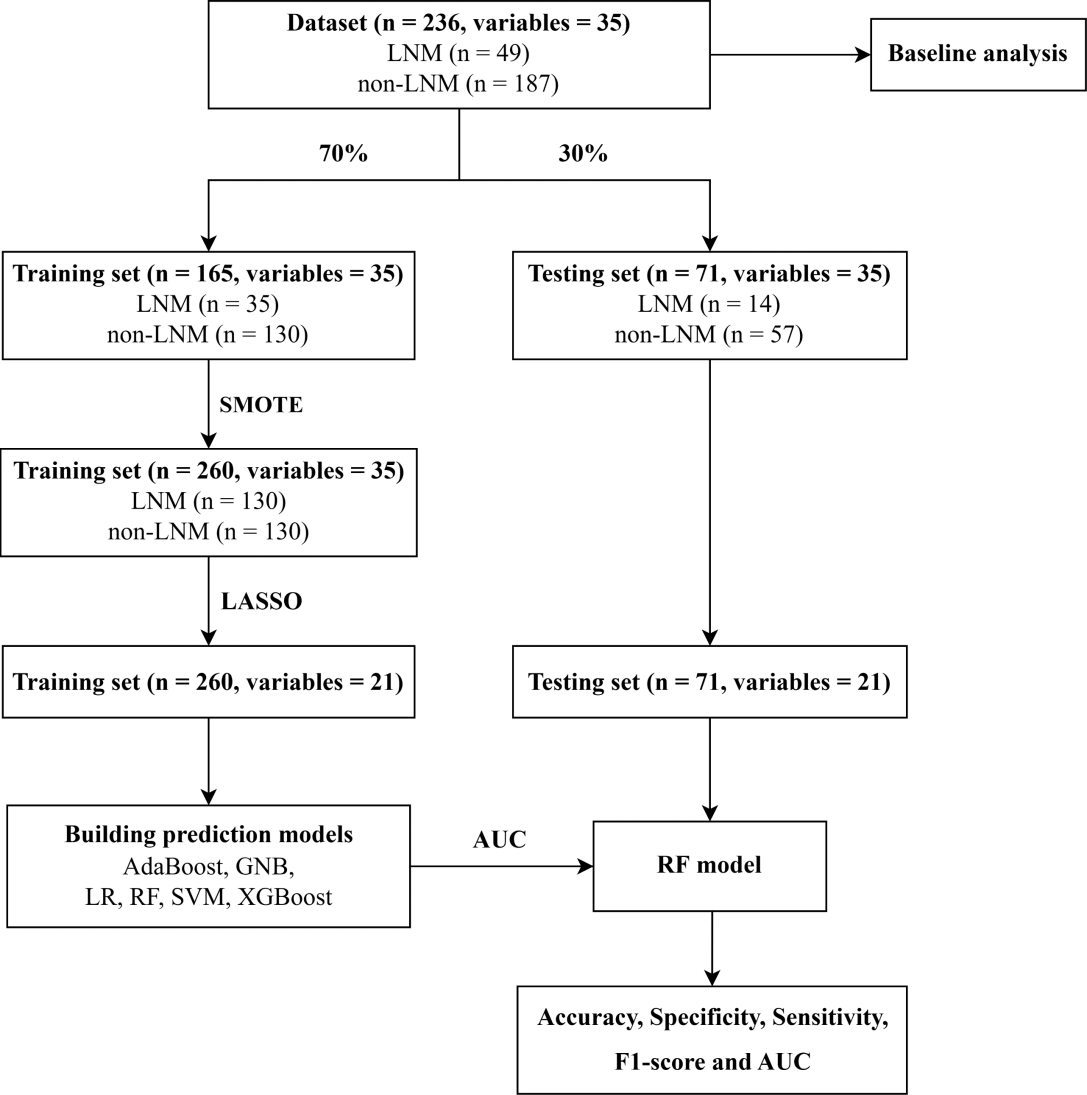
Flowchart for building and validating predictive models. LNM, Lymph node metastasis; SMOTE, Synthetic minority over-sampling technique; LASSO, Least absolute shrinkage and selection operator; AdaBoost, Adaptive Boosting; GNB, Gaussian Naive Bayes; LR, Logistic Regression; RF, Random Forest; SVM, Support Vector Machines; XGBoost, Extreme Gradient Boosting; AUC, Area under receiver operating characteristic curve.

Python (version 3.9) was used to build and verify machine learning models. The flowchart for building and validating machine learning models was shown in **Figure 1**.

### Statistical analysis

There are three main types of data representation: mean ± standard deviation (SD) for normal continuous data, median [interquartile range (IQR)] for non-normal continuous data, and count (percentages) for counting data. The Shapiro-Wilk test was used to examine the normality distribution of the continuous data. For the comparison of all variables between CC patients with and without LNM, the independent sample t test and the Mann-Whitney U test were used to analyze the normal and non-normal continuous data, respectively, and the chi-square test was used for analyzing the counting data. The DeLong test was used to compare the differences between the ROC curves of the six machine learning models (33). Statistical analysis was performed by using SPSS Statistics 26.0 (IBM Corp., Chicago, Illinois, United States of America) software and MedCalc 20.1.0 (Solvusoft., Las Vegas, Nevada, United States of America) software. *P* values less than 0.05 (*P* < 0.05) were considered to be statistically significant.

## Results

### Participant characteristics

The clinical characteristics of the CC patients are shown in **Table 1**. A total of 236 patients with CC were enrolled in this study, and the mean age and body mass index (BMI) of the patients were 53.6 ± 10.5 years and 24.7 ± 3.1 kg/m^2^, respectively. All of the CC patients were classified into two groups (LNM group, n = 49; non-LNM group, n = 187) according to the results of histopathological examinations. The results of the independent sample t test and the chi-square test showed that there were no significant differences in age, BMI, menopausal status, tubal ligation, diabetes, hypertension, histological subtypes of cervical cancer and lymphovascular space invasion between the LNM group and non-LNM group (*P* > 0.05). There was a significant difference in FIGO staging between the LNM group and Non-LNM group (*P* < 0.05).

**Table 1:** Clinical characteristics of cervical cancer patients.

Characteristics	Total (N = 236)	LNM (N = 49)	Non-LNM (N = 187)	*P* value
**Age** (years)	53.6 ± 10.5	54.5 ± 10.3	53.3 ± 10.6	0.501
**BMI** (kg/m^2^)	24.7 ± 3.1	25.1 ± 2.6	24.6 ± 3.3	0.268
**Menopausal status**				0.590
Premenopausal	98 (41.5%)	22 (44.9%)	76 (40.6%)	
Postmenopausal	138 (58.5%)	27 (55.1%)	111 (59.4%)	
**Tubal ligation**				0.800
Negative	129 (54.7%)	26 (53.1%)	103 (55.1%)	
Positive	107 (45.3%)	23 (46.9%)	84 (44.9%)	
**Diabetes**				0.829
Negative	225 (95.3%)	47 (95.9%)	178 (95.2%)	
Positive	11 (4.7%)	2 (4.1%)	9 (4.8%)	
**Hypertension**				0.379
Negative	192 (81.4%)	42 (85.7%)	150 (80.2%)	
Positive	44 (18.6%)	7 (14.3%)	37 (19.8%)	
**FIGO stage**				**<0.001**
I	133 (56.4%)	15 (30.6%)	118 (63.1%)	
II	87 (36.8%)	20 (40.8%)	67 (35.8%)	
III	16 (6.8%)	14 (28.6%)	2 (1.1%)	
IV	0 (0%)	0 (0%)	0 (0%)	
**LVSI**				1.000
Negative	3 (1.3%)	0 (0%)	3 (1.6%)	
Positive	233 (98.7%)	49 (100%)	184 (98.4%)	
**Histological subtypes**				0.163
adenocarcinoma	28 (11.9%)	3 (6.1%)	25 (13.4%)	
SCC	208 (88.1%)	46 (93.9%)	162 (86.6%)	

Values are expressed as the number of patients (percentages) or mean ± SD. *P* values refer to the results of independent samples t test and chi-square test.

N, number of individuals; LNM, lymph node metastasis; BMI, body mass index; FIGO, International Federation of Gynecology and Obstetrics; LVSI, lymphovascular space invasion; SCC, squamous cell carcinoma.

**Table 2** shows the basic descriptive statistics of the hematological indicators in CC patients, as well as the results of the independent sample t test and the Mann-Whitney U test. In the univariate analyses, 8 hematological indicators, including SCC-Ag, DD, HGB, PAB, TP, ALB, TCHO, and LDL, were significantly different between the LNM group and the non-LNM group (*P* < 0.05). These results were based on the raw data analysis of 236 CC patients, whereas the feature selection was based on the processed data. Class balancing of the training data by using SMOTE resulted in 130 CC patients with LNM and 130 CC patients without LNM in the training set.

**Table 2:** Association between hematologic indicators and lymph node metastasis status.

Characteristics	Total (N = 236)	LNM (N = 49)	Non-LNM (N = 187)	*P* value
SCC-Ag	1.70 [3.93]	6.00 [12.83]	1.40 [2.71]	**<0.001**
PT	10.83 ± 0.59	10.79 ± 0.57	10.84 ± 0.60	0.605
PT-INR	0.94 ± 0.05	0.93 ± 0.05	0.94 ± 0.05	0.318
PTA	111.55 ± 10.78	110.95 ± 10.89	111.71 ± 10.77	0.661
TT	18.27 ± 1.34	18.35 ± 1.59	18.25 ± 1.26	0.703
APTT	25.24 ± 2.12	24.94 ± 2.03	25.31 ± 2.14	0.270
FIB	2.93 ± 0.79	2.83 ± 0.71	2.95 ± 0.81	0.349
DD	0.33 [0.32]	0.47 [0.49]	0.32 [0.25]	**0.003**
WBC	5.92 ± 1.73	5.55 ± 1.39	6.01 ± 1.80	0.092
NEUT	3.23 [1.59]	3.03 [2.04]	3.26 [1.47]	0.222
LYM	1.84 ± 0.59	1.72 ± 0.54	1.87 ± 0.61	0.117
MON	0.40 [0.15]	0.41 [0.13]	0.40 [0.16]	0.359
NEUT %	58.22 ± 9.34	58.29 ± 10.38	58.21 ± 9.08	0.959
LYM %	31.96 ± 8.99	32.02 ± 9.86	31.94 ± 8.79	0.955
MON %	7.16 ± 2.90	7.35 ± 1.85	7.11 ± 3.12	0.613
HGB	121.21 ± 14.49	117.00 ± 16.17	122.31 ± 13.86	**0.022**
PLT	243.90 ± 65.46	241.86 ± 61.43	244.44 ± 66.63	0.807
MPV	10.80 [1.48]	10.70 [1.65]	10.80 [1.50]	0.423
PCT	0.26 ± 0.07	0.26 ± 0.07	0.26 ± 0.07	0.652
PDW	12.70 [2.88]	12.50 [2.90]	12.70 [2.90]	0.429
P-LCR	32.14 ± 9.21	31.11 ± 9.75	32.41 ± 9.07	0.378
ALT	14.00 [5.00]	14.00 [6.00]	14.00 [5.00]	0.475
AST	20.06 ± 11.41	21.22 ± 14.36	19.76 ± 10.52	0.425
PAB	264.15 ± 59.61	246.35 ± 48.24	268.81 ± 61.51	**0.019**
TP	68.39 ± 4.99	67.00 ± 4.82	68.76 ± 4.99	**0.028**
ALB	42.31 ± 3.46	41.30 ± 3.00	42.57 ± 3.53	**0.022**
GLB	26.08 ± 4.57	25.69 ± 4.69	26.18 ± 4.55	0.508
A/G	1.60 [0.50]	1.60 [0.35]	1.60 [0.50]	0.892
TCHO	4.30 ± 0.91	4.01 ± 0.76	4.38 ± 0.93	**0.011**
TG	1.11 [0.91]	1.12 [0.80]	1.11 [0.98]	0.830
HDL	1.13 ± 0.26	1.07 ± 0.23	1.14 ± 0.26	0.101
LDL	2.57 ± 0.64	2.41 ± 0.54	2.61 ± 0.66	**0.032**
Cys C	0.80 [0.20]	0.80 [0.23]	0.80 [0.20]	0.994
CPR	1.60 [2.21]	1.50 [2.66]	1.60 [2.20]	0.643
SOD	170.97 ± 24.48	166.20 ± 17.46	172.22 ± 25.90	0.126

Values are expressed as mean ± SD or median [interquartile range (IQR)]. *P* values refer to the results of independent samples t-test or Mann-Whitney U test. Bold values indicates a P value of less than 0.05.

N, number of individuals; LNM, lymph node metastasis; SCC-Ag, squamous cell carcinoma antigen; PT, prothrombin time; PT-INR, international normal ratio of prothrombin time; PTA, prothrombin activity; TT, thrombin time; APTT, activated partial thromboplastin time; FIB, fibrinogen; DD, D-dimer; WBC, white blood cell; NEUT, neutrophils; LYM, lymphocytes; MON, monocytes; NEUT %, percentage of neutrophils; LYM %, percentage of lymphocytes; MON %, percentage of monocytes; HGB, hemoglobin; PLT, platelets; MPV, mean platelet volume; PCT, the product of MPV and PLT; PDW, platelet distribution width; PLCR, platelet large cell ratio; ALT, alanine aminotransferase; AST, aspartate aminotransferase; PAB, prealbumin; TP, total protein; ALB, albumin; GLB, globulin; A/G, albumin to globulin ratio; TCHO, total cholesterol; TG, triglyceride; HDL, high-density lipoprotein; LDL, low-density lipoprotein; Cys C, cystatin C; CRP, c-reactive protein; SOD, superoxide dismutase.

### Feature selection

**Figure 2:**
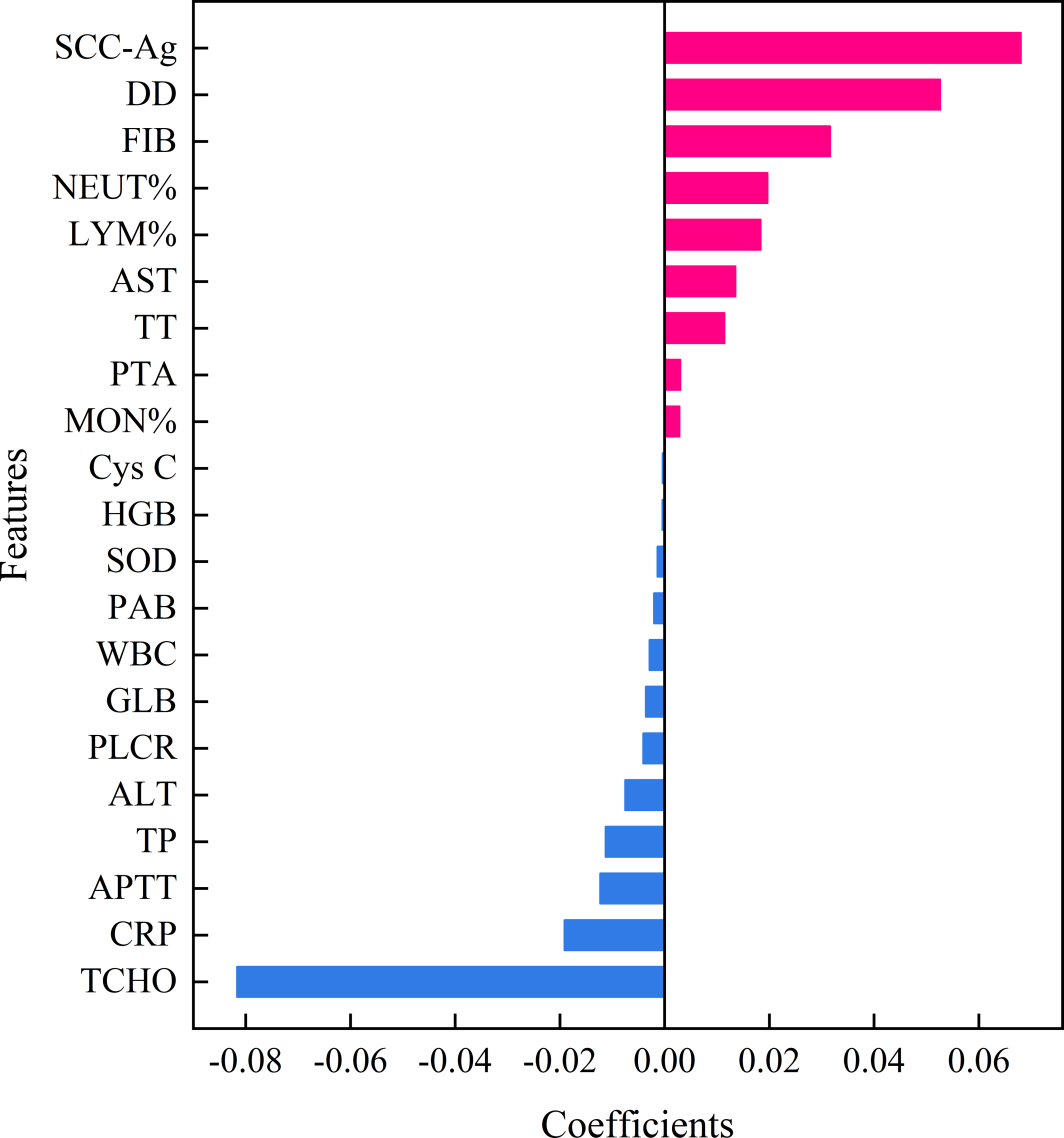
Features selected by LASSO with their estimated Coefficients. SCC-Ag, squamous cell carcinoma antigen; PT-INR, International normal ratio of prothrombin time; TT, thrombin time; APTT, activated partial thromboplastin time; FIB, fibrinogen; DD, D-Dimer; WBC, white blood cell; NEUT %, percentage of neutrophil; LYM %, percentage of lymphocyte; MON %, percentage of monocytes; HGB, hemoglobin; PLCR, platelet large cell ratio; ALT, alanine aminotransferase; AST, aspartate aminotransferase; PAB, prealbumin; TP, total protein; GLB, globulin; TCHO, total cholesterol; Cys C, Cystatin C; CRP, c-reactive protein; SOD, superoxide dismutase; LASSO, Least absolute shrinkage and selection operator.

In this study, the LASSO feature selection technique was applied to select 21 features from the 35 features in the training dataset. **Figure 2** illustrated the features selected by LASSO and their estimated coefficients. The top 21 hematological indicators in terms of coefficients (from high to low) were TCHO, SCC-Ag, DD, FIB, NEUT %, CRP, LYM %, AST, APTT, TT, TP, ALT, PLCR, GLB, PTA, WBC, MON %, PAB, SOD, HGB and Cys C. The absolute value of coefficients reflects the feature importance of hematological indicators.

### Establishment and evaluation of machine learning models

**Figure 3:**
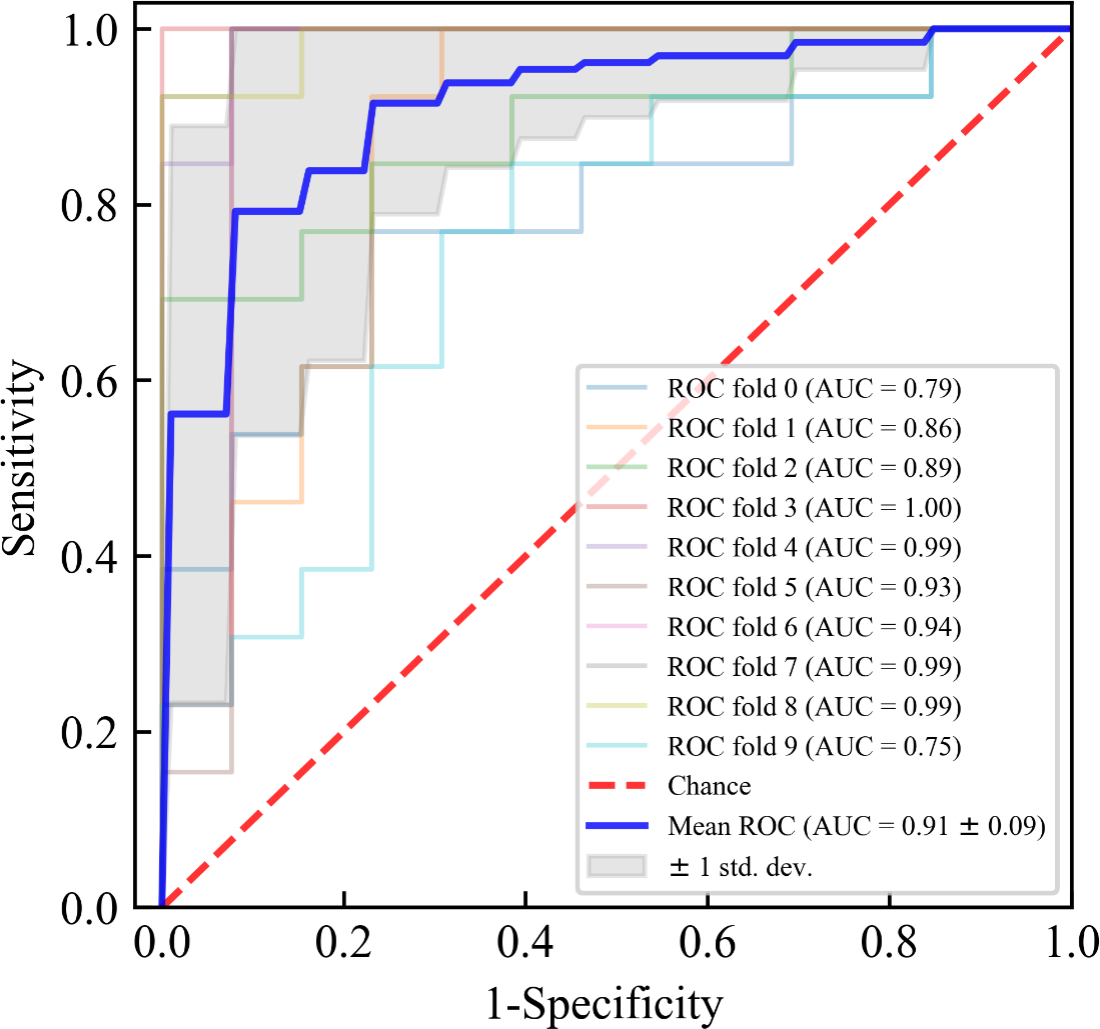
Receiver operating characteristic (ROC) curves for ten-fold cross-validation of the Random Forest (RF) model in the training set. The blue solid line represents the average ROC curve with ten-fold cross-validation. The red diagonal line denotes an area under the ROC curve (AUC) of 0.5, which represents a random probability (P = 0.5). The shaded area around the average ROC curve reflects the 95% confidence interval. AUC, area under receiver operating characteristic curve.

The results of ten-fold cross-validation in the training set show that the RF model outperforms the other five machine learning models (including AdaBoost, GNB, LR, SVM, and XGBoost) in all predictive indicators (**Table 3**). The specific performance indicators of the RF model were AUC (0.910, 95% confidence interval [CI]: 0.820–1.000) (**Figure 3**), accuracy (0.831, 95% CI: 0.702–0.960), specificity (0.835, 95% CI: 0.708–0.962), sensitivity (0.831, 95% CI: 0.702–0.960), and F1-score (0.829, 95% CI: 0.696–0.962).

**Table 3:** Ten-fold cross-validated predictive performance of the six models in the training set.

Model	Accuracy	Specificity	Sensitivity	F1-score	PPV	NPV	AUC
AdaBoost	0.785 (0.145)	0.797 (0.147)	0.785 (0.145)	0.782 (0.146)	1.000 (0.000)	0.992 (0.000)	0.831 (0.130)
GNB	0.611 (0.081)	0.687 (0.101)	0.612 (0.081)	0.558 (0.104)	0.794 (0.024)	0.793 (0.034)	0.786 (0.122)
LR	0.719 (0.097)	0.723 (0.100)	0.719 (0.097)	0.718 (0.098)	0.796 (0.024)	0.760 (0.023)	0.793 (0.102)
RF	0.831 (0.129)	0.835 (0.127)	0.831 (0.129)	0.829 (0.133)	1.000 (0.000)	0.986 (0.007)	0.910 (0.090)
SVM	0.719 (0.100)	0.731 (0.107)	0.719 (0.100)	0.716 (0.101)	0.663 (0.011)	0.763 (0.024)	0.782 (0.124)
XGBoost	0.826 (0.129)	0.832 (0.129)	0.827 (0.129)	0.826 (0.129)	1.000 (0.000)	0.992 (0.000)	0.901 (0.107)

Values are expressed as mean (standard deviation).

AdaBoost, Adaptive Boosting; GNB, Gaussian Naive Bayes; LR, Logistic Regression; RF, Random Forest; SVM, Support Vector Machines; XGBoost, Extreme Gradient Boosting; PPV, Positive Predictive Value; NPV, Negative Predictive Value; AUC, Area under receiver operating characteristic curve.

**Figure 4:**
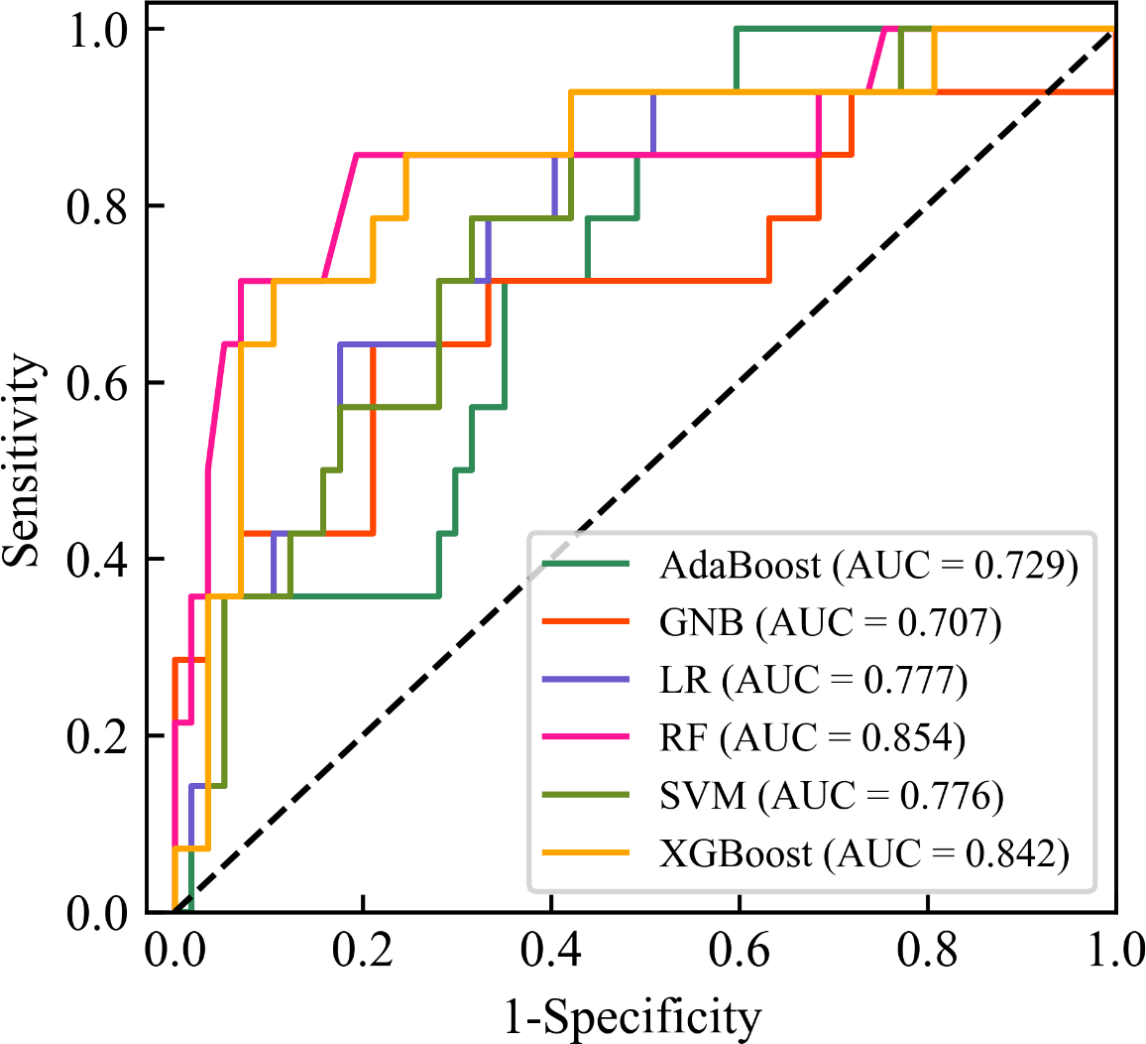
Receiver operating characteristic (ROC) curves of six machine learning models for predicting lymph node metastasis of cervical cancer on the testing set. The black diagonal line denotes an area under the ROC curve (AUC) of 0.5, which represents a random probability (P = 0.5). AdaBoost, Adaptive Boosting; GNB, Gaussian Naive Bayes; LR, Logistic Regression; RF, Random Forest; SVM, Support Vector Machines; XGBoost, Extreme Gradient Boosting; AUC, Area under receiver operating characteristic curve.

**Figure 4** showed the ROC curves of six machine learning models for predicting CC LNM on the testing set. Among them, RF had the highest AUC value (AUC = 0.854), which was significantly higher than the other five models (all *P* values < 0.05, Delong test), which was a key metric for assessing the performance of predictive models. In the testing set, the accuracy, specificity, sensitivity, F1-score, and AUC of the RF model were all above 0.8, and the RF model showed the best performance among the six machine learning algorithms (**Table 4**). Therefore, the RF model was determined to be the best model in this study.

**Table 4:** Predictive performance of six machine learning models in the testing set.

Model	Accuracy	Specificity	Sensitivity	F1score	PPV	NPV	AUC
AdaBoost	0.577	0.491	0.929	0.642	0.309	0.966	0.729
GNB	0.761	0.789	0.643	0.709	0.429	0.900	0.707
LR	0.789	0.825	0.643	0.723	0.474	0.904	0.777
RF	0.817	0.807	0.857	0.831	0.523	0.958	0.854
SVM	0.648	0.579	0.929	0.713	0.351	0.971	0.776
XGBoost	0.774	0.754	0.857	0.802	0.500	0.957	0.842

AdaBoost, Adaptive Boosting; GNB, Gaussian Naive Bayes; LR, Logistic Regression; RF, Random Forest; SVM, Support Vector Machines; XGBoost, Extreme Gradient Boosting; PPV, Positive Predictive Value; NPV, Negative Predictive Value; AUC, Area under receiver operating characteristic curve.

## Discussion

In this study, six machine learning models were used to predict LNM status in CC patients. The machine learning models were based on a variety of preoperative hematological indicators, including routine blood indicators, routine biochemical indicators, coagulation function indicators, and tumor markers. The results of ten-fold cross-validation showed that the overall prediction performance of the RF model was better than that of the other five models, thus indicating that the model had the best stability.

In recent years, ML techniques have been widely used to identify LNM in CC patients. For example, Liu et al. (34) collected clinical features and MRI radiomics features of 180 CC patients and established 7 ML models. The results showed that among the 7 ML models, Multinomial Naive Bayes (MNB) had the most robust predictive performance, with an AUC of 0.745, an accuracy of 0.778, and a specificity of 0.900. Compared to the present study, the model needs to be improved in terms of accuracy of prediction, and the method is more costly and time-consuming to test. Guan et al. (35) collected preoperative 5-minute electrocardiograms from 292 CC patients and developed 6 ML models based on 32 heart rate variability parameters. The results showed that among the 6 ML models, the RF model had the best predictive performance (AUC of 0.852, accuracy of 0.744, sensitivity of 0.783 and specificity of 0.785). In contrast, the RF model characterized by hematological parameters in this study showed improved AUC, accuracy, sensitivity and specificity (AUC of 0.854, accuracy of 0.817, sensitivity of 0.857 and specificity of 0.807).

To improve the interpretability of machine learning models, we used coefficients to represent the feature importance of each hematological indicator. Higher feature importance indicates that the feature is more useful for predicting CC LNM. In this study, TCHO showed the highest feature importance. Increased serum TCHO levels have been reported to be a risk factor for the development of certain cancers, and serum TCHO levels have been associated with LNM in a variety of cancers, such as esophageal cancer, gastric cancer and pancreatic cancers. Sako et al. (36) found that TCHO levels in esophageal cancer patients with LNM were significantly higher than those without LNM. Wu et al. (37) demonstrated that TCHO levels in pancreatic cancer patients were significantly correlated with tumor grade and LNM. Kitayama et al. (38) reported that patients with early gastric cancer who suffered from hypercholesterolemia (TCHO ≥ 220 mg/dl) had a significantly higher rate of LNM. It has been shown that T lymphocytes play a major role in killing malignant cells, but their activity is influenced by the tumor microenvironment. High cholesterol levels upregulate the expression of immune checkpoints in T lymphocytes, which leads to a weakening of the anti-tumor function of T cells (39). In addition, Mahmoud et al. (40) found that prostate cancer cells store cholesterol and use it as energy for growth. Therefore, it is possible that elevated levels of TCHO promote malignant tumor growth and thus malignant tumorigenesis LNM. To the best of our knowledge, there are no studies on the correlation between LNM and TCHO in CC. The results of this study confirmed that TCHO levels in CC patients were significantly correlated with LNM. However, the exact mechanism of TCHO as a predictor of LNM in CC patients is unclear and requires further study.

In this study, SCC-Ag was ranked second in terms of feature importance. SCC-Ag is a specific antigen produced by squamous cell carcinoma (SCC) that has good application value for predicting LNM in cervical cancer derived from squamous cells (41, 42). Preoperative serum SCC-Ag is the tumor marker that is commonly used to predict squamous cell CC LNM (43, 44). Previous studies have suggested that preoperative high SCC-Ag levels may be associated with CC LNM (45–47). Wei et al. (48) found that cancer-associated fibroblasts (CAFs) in patients with cervical squamous cell carcinoma impaired lymphatic endothelial barriers by activating the integrin-FAK/Src-VE-cadherin signaling pathway in lymphatic endothelial cells, thus consequently enhancing CC LNM.

In this study, coagulation function indicators (such as DD and FIB) also showed high feature importance. Previous studies have indicated that the coagulation function of patients with malignant tumors exhibit different degrees of abnormality (49–51). This may be related to tumor cells causing changes in coagulation function through various pathways to promote tumor growth, infiltration, and metastasis (52). Similarly, the hyperactivation of the coagulation system in CC patients can promote LNM development (53, 54). In this study, the univariate analysis confirmed that the DD levels of CC patients with LNM were significantly higher than those of CC patients without LNM (P = 0.003). Remarkably, in our study, hematological indicators such as TT, APTT, PT-INR, TP, and NEUT% were also confirmed to contribute to the construction of machine learning models. However, the specific mechanism of the above-mentioned indicators as predictors of LNM in CC patients is unclear, and further studies are warranted. Furthermore, it has been suggested that some hematological parameters that were not used in this study, such as sugar chain antigen 125 (CA125), sugar chain antigen 199 (CA19-9), α fetoprotein (AFP), and alkaline phosphatase (ALP), may also be associated with LNM in CC patients, which may also provide a feasible direction for future research (55).

However, this study also had some limitations. First, the present study was a retrospective analysis derived from a single- center, and a relatively small sample size was taken into account. Therefore, further validation of predictive models will need to be conducted in a larger multicenter study to establish the robustness of the current findings. Second, hematological indicators are always affected to varying degrees by testing equipment and testing reagents. Thus, hematological indicators will need to be collected under different conditions in the future to verify the generalizability of the predictive model. Third, CC often occurs in remote areas with limited medical care, leading to some difficulties in collecting the required hematological parameters (e.g., SCC-Ag). In the future, there will be a need to use fewer hematological indicators for modeling while ensuring the performance of the ML model to improve the usability of the ML model in most areas.

## Conclusion

In conclusion, we used machine learning algorithms to establish six machine learning models based on preoperative hematological indicators for the preoperative prediction of LNM status in CC patients. Ten-fold cross-validation proved that the RF model had higher stability. The higher AUC values of the RF model in the testing set indicate a better generalization performance. Our results suggested that the RF model based on preoperative hematological indicators had great potential in clinical practice. Through further validation and refinement, the RF model has the potential to help develop more effective treatment plans for cervical cancer patients through preoperative diagnosis.

## Data Availability

The original contributions presented in the study are included in the article/supplementary material. Further inquiries can be directed to the corresponding authors.
